# Distinct effects of TRAIL on the mitochondrial network in human cancer cells and normal cells: role of plasma membrane depolarization

**DOI:** 10.18632/oncotarget.4268

**Published:** 2015-05-25

**Authors:** Yoshihiro Suzuki-Karasaki, Kyoko Fujiwara, Kosuke Saito, Miki Suzuki-Karasaki, Toyoko Ochiai, Masayoshi Soma

**Affiliations:** ^1^ Division of Physiology, Department of Biomedical Sciences, Nihon University School of Medicine, Tokyo, Japan; ^2^ Innovative Therapy Research Group, Nihon University Research Institute of Medical Science, Tokyo, Japan; ^3^ Division of General Medicine, Department of Internal Medicine, Nihon University School of Medicine, Tokyo, Japan; ^4^ Department of Dermatology, Nihon University Surugadai Hospital, Tokyo, Japan

**Keywords:** TRAIL, mitochondrial fragmentation, depolarization, reactive oxygen species, tumor-selective killing

## Abstract

Apo2 ligand/tumor necrosis factor-related apoptosis-inducing ligand (Apo2L/TRAIL) is a promising anticancer drug due to its tumor-selective cytotoxicity. Here we report that TRAIL exhibits distinct effects on the mitochondrial networks in malignant cells and normal cells. Live-cell imaging revealed that multiple human cancer cell lines and normal cells exhibited two different modes of mitochondrial responses in response to TRAIL and death receptor agonists. Mitochondria within tumor cells became fragmented into punctate and clustered in response to toxic stimuli. The mitochondrial fragmentation was observed at 4 h, then became more pronounced over time, and associated with apoptotic cell death. In contrast, mitochondria within normal cells such as melanocytes and fibroblasts became only modestly truncated, even when they were treated with toxic stimuli. Although TRAIL activated dynamin-related protein 1 (Drp1)-dependent mitochondrial fission, inhibition of this process by Drp1 knockdown or with the Drp1 inhibitor mdivi-1, potentiated TRAIL-induced apoptosis, mitochondrial fragmentation, and clustering. Moreover, mitochondrial reactive oxygen species (ROS)-mediated depolarization accelerated mitochondrial network abnormalities in tumor cells, but not in normal cells, and TRAIL caused higher levels of mitochondrial ROS accumulation and depolarization in malignant cells than in normal cells. Our findings suggest that tumor cells are more prone than normal cells to oxidative stress and depolarization, thereby being more vulnerable to mitochondrial network abnormalities and that this vulnerability may be relevant to the tumor-targeting killing by TRAIL.

## INTRODUCTION

Apo2 ligand/tumor necrosis factor-related apoptosis-inducing ligand (Apo2L/TRAIL) is a member of the tumor necrosis factor cytokine superfamily, which has emerged as a promising anticancer drug, because it induces apoptosis in cancer cells with minimal cytotoxicity toward normal cells [1–4]. TRAIL binds to two death receptors (DRs), TRAIL receptor (TRAIL-R)1/DR4 and TRAIL-R2/DR5 to trigger the extrinsic and intrinsic apoptotic pathways [5, 6]. However, multiple cancer cell types such as malignant melanoma, glioma, and non-small cell lung cancer (NSCLC) cells are resistant to TRAIL treatment despite expressing DRs on their cell surface. Moreover, TRAIL-responsive tumors acquire a resistant phenotype that renders TRAIL therapy ineffective [7, 8]. Therefore, overcoming TRAIL resistance is necessary for effective TRAIL therapy, and small molecules that can potentiate TRAIL effectiveness are urgently required.

Persistent depolarization of the plasma membrane potential is an early event essential for apoptosis and caspase-3 activation in human malignant cells that is induced by diverse pro-apoptotic agents including anti-Fas antibody, arsenic trioxide, and the mitochondrial toxin rotenone [9–11]. We previously showed that depolarization is an early and prerequisite event during TRAIL-induced apoptosis in malignant tumor cells such as melanoma, leukemia, and NSCLC. TRAIL dose- and time-dependently induces robust depolarization in these cells after a time lag of 2−4 h [12, 13]. Moreover, persistent depolarization induced by high K^+^ loading or ATP-sensitive K^+^ channel inhibitors sensitizes melanoma cells. This sensitization is associated with increased intrinsic and endoplasmic reticulum death pathways. A number of pro-apoptotic responses, including mitochondrial reactive oxygen species (mROS) generation, collapse of mitochondrial membrane potential (MMP), and oxidation of cardiolipin within mitochondria are potentiated. All of these events are known to cooperatively lead to mitochondrial membrane integrity disruption, a gate keeping player to the release of pro-apoptotic proteins such as cytochrome c. In contrast, TRAIL and membrane-depolarizing agents alone or in combination minimally induce apoptosis in normal melanocytes, though the cells express DR4 and DR5 on their surfaces. These observations suggest that malignant cells are more prone than normal cells to the depolarization-triggered apoptosis. However, the precise mechanisms by which depolarization potentiates mitochondrial dysfunction remain unclear.

Mitochondria are highly dynamic organelles with a reticular network that is regulated by the balance between fission and fusion. Mitochondrial morphology is critical for cell function and apoptosis [14, 15]. The mitochondrial network depends on the delicate balance between two antagonistic machineries responsible for fission and fusion of the mitochondrial membrane. Mitochondrial network dynamics is controlled by dynamin-related proteins with GTPase activity, namely mitofusin 1/2 (Mfn1/2), optic atrophy 1 (OPA1), and dynamin-related protein 1 (Drp1). Mfn1/2, and OPA1 act in concert to regulate mitochondrial fusion and cristae organization, while Drp1 regulates mitochondrial fission [16, 17]. A well-balanced fission and fusion is required for healthy mitochondria, because either process is essential for cell function and survival. However, the role of the mitochondrial network in cancer cell apoptosis is a matter of debate, because controversial results have been reported about the role of mitochondrial fission. Mitochondrial fission is thought to be essential for mitochondrial outer membrane permeabilization and cytochrome c release [18]. However, an increasing body of evidence suggests that mitochondrial fission is pro-apoptotic or anti-apoptotic, depending on the cell type and the applied apoptotic stimuli [19–24].

In an attempt to elucidate the role of mitochondrial fission in cancer cell apoptosis, we previously studied the effect of enforced mitochondrial fusion due to Drp1 inhibition, on TRAIL-induced apoptosis. Both Drp1 knockdown and the Drp1 inhibitor, mitochondrial division inhibitor-1 (mdivi-1) [25], inhibits mitochondrial fission, and kills and sensitizes cancer cells to the apoptosis [26]. These effects are generally observed among different cancer cell lines. Moreover, the sensitization is associated with increased intrinsic pathway activity and is preceded by depolarization, MMP collapse, mROS generation, and cardiolipin oxidation. In contrast, mdivi-1 and TRAIL, alone or in combination induced minimal apoptosis in normal melanocytes and fibroblasts. These observations suggest that mitochondrial fission acts as an anti-apoptotic factor in a tumor-specific manner. These findings led us to investigate the possible role of the mitochondrial network in TRAIL-induced cancer cell apoptosis. Here we report, for the first time, that TRAIL induces pro-apoptotic mitochondrial network abnormalities in human malignant cells, but not in normal cells. We also demonstrated that this difference is attributed to the distinct sensitivities of these cells to ROS and depolarization, which are required for the pro-apoptotic mitochondrial network abnormalities.

## RESULTS

### TRAIL induces punctate mitochondria and their clustering in human cancer cells, but not in normal cells

**Figure 1:**
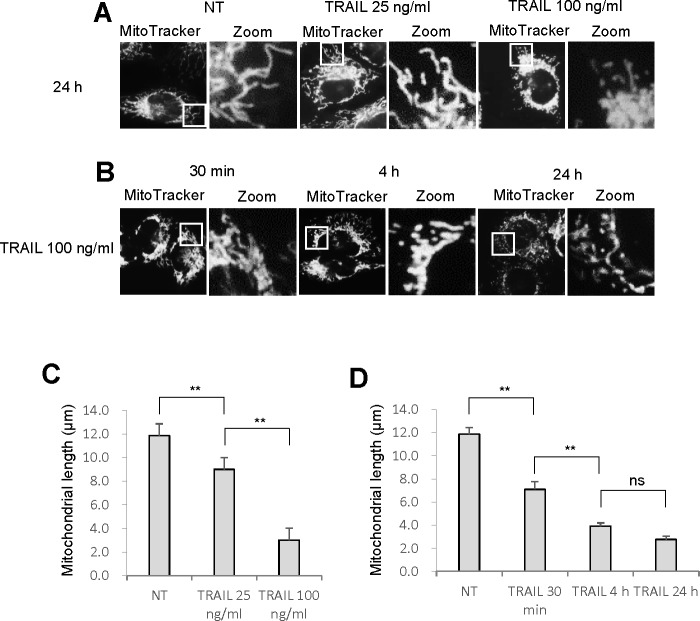
TRAIL modulates the mitochondrial network in melanoma cells **A.**, **B.** A375 melanoma cells in FBS/DMEM were plated on a chambered coverglass and treated with soluble recombinant human TRAIL (25, 100 ng/ml) for 24 h **A.** or TRAIL (100 ng/ml) for 30 min, 4 h, or 24 h **B.** at 37°C. Then, the cells were washed, stained with MitoTracker Red for 1 h, washed, and analyzed for mitochondrial network. For each sample, pictures of three different visual fields (totally ∼ 40 cells in a single sample) were randomly analyzed for the average mitochondrial length using NIH ImageJ software. **C.**, **D.** Statistical analyses of the average mitochondrial length for experiment A and B, respectively. The values represent the means ± SE of three or four independent experiments. Data were analyzed by one-way analysis of variance followed by the post-hoc Tukey test. **P < 0.01; ns, not significant.

**Figure 2:**
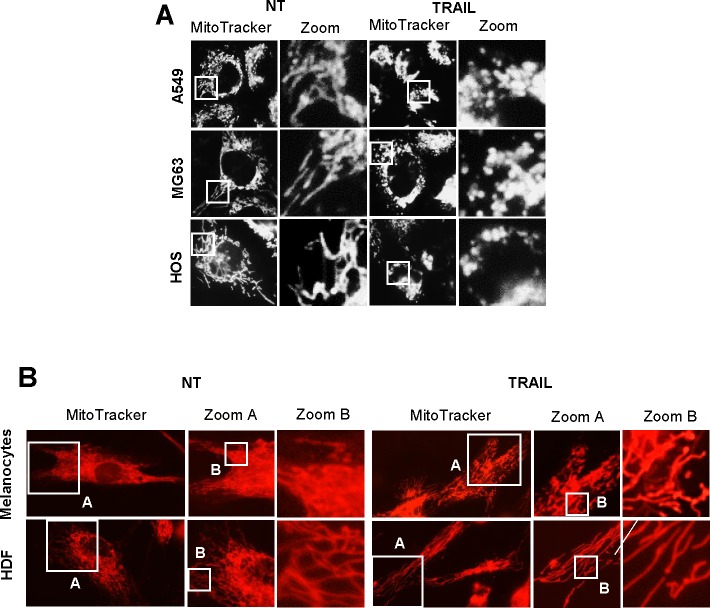
TRAIL induces mitochondrial fragmentation and clustering in multiple cancer cell lines, but not in normal cells **A.** A549 NSCLC cells (top panels), MG63 (middle panels) and HOS osteorsarcoma cells (bottom panels) were treated with TRAIL (100 ng/ml) for 24 h at 37°C. **B.** Normal melanocytes and human dermal fibroblasts (HDF) were treated with TRAIL (100 ng/ml) for 24 h at 37°C.

As reported earlier [12], TRAIL dose-dependently increased apoptotic cell death in A375 melanoma cells. Until 24 h, treatment with up to 100 ng/ml of TRAIL induced a moderate (<30%) increase in the number of annexin V-positive cells. On the other hand, 72 h of treatment with 25 ng/ml of TRAIL caused a modest increase in the number of annexin V-positive cells (18.2 ± 0.7 %, *n* = 4) while treatment with 100 ng/ml of TRAIL substantially increased the cell population (59.8 ± 2.9 %, *n* = 4). Therefore, we used 25 ng/ml and 100 ng/ml TRAIL, respectively as a weak and strong inducer of apoptosis throughout the present study. Then, we determined whether TRAIL affected mitochondrial network dynamics in these cells. The cells were treated with recombinant human TRAIL for various time periods, stained with the mitochondria-targeting dye MitoTracker Red CMXRos, and then their mitochondrial network were analyzed using a cell imaging system equipped with digital inverted microscope. In control cells, the mitochondria mainly consisted of a tubular morphology of ∼ 12 μm, a hallmark of well-balanced fission and fusion (Figure 1A, left). TRAIL treated cells showed multiple mitochondrial network abnormalities in a dose- and time-dependent manner. After 24 h of treatment with TRAIL (25 ng/ml), a modest mitochondrial truncation took place (Figure 1A, middle), resulting in short mitochondria of the average length of ∼9 μm (Figure 1C). Upon stimulation with a higher concentration of TRAIL (100 ng/ml), substantial mitochondrial fragmentation occurred (Figure 1A, right), resulting in extremely short mitochondria of the average length of ∼ 3 μm (Figure 1C). The majority of the mitochondria became punctate and clustered. Time course experiments indicated that for TRAIL (100 ng/ml), a modest truncation was observed as rapidly as 30 min, while punctate mitochondria and their clustering were first detected at 4 h and then became more pronounced over time (Figure 1B). Next, we examined whether this phenomenon is specific for melanoma cells or generally observed among multiple cancer cell types. The mitochondria within A549 NSCLC cells exhibited moderately fragmented network even in the absence of stimulus (Figure 2A, top left). After TRAIL treatment, clustering of punctate mitochondria became clear (Figure 2A, top right). Similarly, the mitochondria within two osteosarcoma cell lines MG63 and HOS also became fragmented into punctate and clustered after TRAIL treatment (Figure 2A, middle and bottom). These results show that TRAIL induces similar mitochondrial network abnormalities in different human cancer cell types. Then, we examined whether these mitochondrial network abnormalities are specific for tumor cells. As shown in Figure 2B, TRAIL treatment resulted in modest fission, but not clustering of punctate mitochondria in melanocytes and fibroblasts. These results indicate that TRAIL evokes clustering of punctate mitochondria in a tumor-specific manner.

### The mitochondrial network abnormalities are associated with cell death

**Figure 3:**
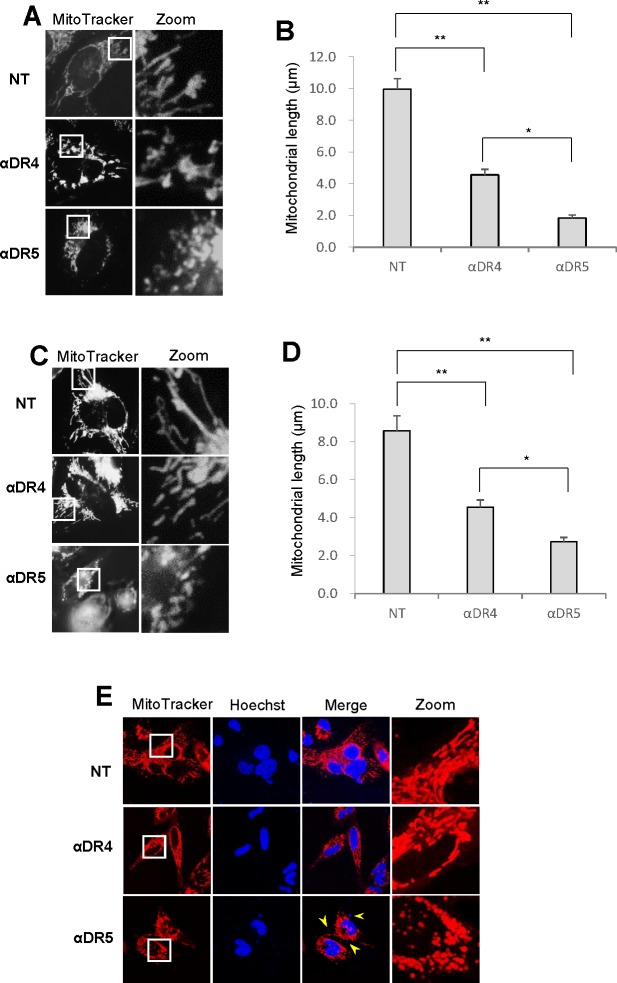
Mitochondrial fragmentation and clustering are specifically associated with apoptotic cell death **A.**, **C.** A375 cells **A.** and A2058 cells **C.** were treated with agonistic anti-DR4 or DR5 (1 μg/ml) for 24 h at 37°C, and analyzed for mitochondrial morphology. **B.**, **D.** Statistical analysis of the average mitochondrial length of A375 cells and A2058 cells, respectively. The values represent the means ± SE of three or four independent experiments. *P < 0.05; **P < 0.01. **E.** Representative confocal imaging in A2058 cells. Mitochondria and nuclei were stained with MitoTracker Red and Hoechst 33342, respectively and observed under a confocal microscope. Arrows indicate fragmented nuclei.

Microscopic analyses showed that healthy cells possess tubular, elongated, or modestly fragmented mitochondria, while morphologically damaged cells regularly harbor punctate and clustered mitochondria. To clarify the possible link between the mitochondrial network abnormalities and cell death, we compared the effects of two different anti-DR4/5 antibodies with different pro-apoptotic activities on mitochondrial network. An anti-DR5 antibody (αDR5; MAB631 1 μg/ml) was equipotent to TRAIL (100 ng/ml) at inducing apoptotic cell death, while an anti-DR4 antibody (αDR4; MAB631) was ineffective [27] (see Figure 5C). Treatment with αDR4 resulted in modest fragmentation of mitochondria in A375 cells (Figure 3A, middle), while treatment with αDR5 resulted in considerable increases in punctate and clustered mitochondria (Figure 3A, bottom). Similar distinct effects of αDR4 and αDR5 on mitochondrial morphology were observed in A2058 cells (Figure 3C). These observations were confirmed by mitochondrial length measurements (Figure 3B, 3D) as well as confocal imaging (Figure 3E). These cells harboring heavily fragmented and clustered mitochondria possessed brighter, fragmented nuclei, indicating the onset of nuclear fragmentation and chromatin condensation, hallmarks of apoptosis.

### TRAIL induces Drp1-dependent mitochondrial fission and its inhibition accelerates the mitochondrial network abnormalities

**Figure 4:**
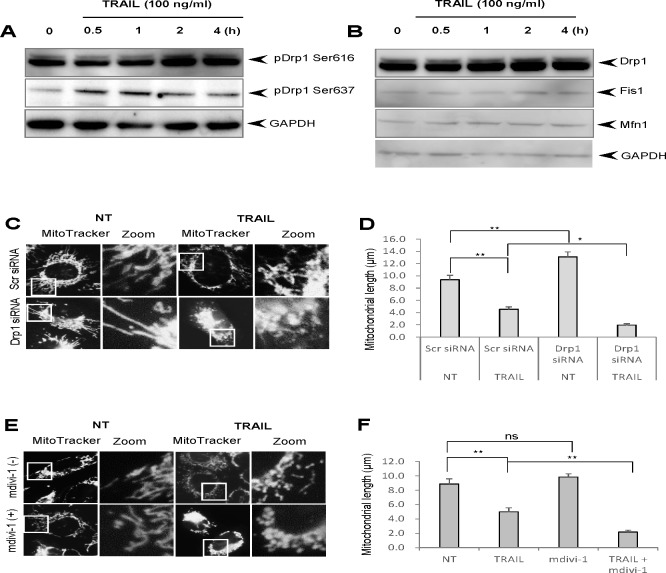
TRAIL induces Drp1-dependent mitochondrial fission whose inhibition accelerates the pro-apoptotic mitochondrial network abnormalities **A.**, **B.** A375 cells were treated with TRAIL (100 ng/ml) for the indicated time periods and analyzed for their expression of pDrp1 Ser616 and Ser637 **A.** or Drp1, Fis1, and Mfn1 **B.** using immunoblotting. GAPDH was used as a loading control. **C.**, **E.** Control and Drp1 knockdown A375 cells **C.** were treated with TRAIL (100 ng/ml) or A375 cell were treated with TRAIL (100 ng/ml) alone or in combination with mdivi-1 (50 μM) **E.** for 24 h at 37°C, and analyzed for mitochondrial morphology. **D.**, **F.** Statistical analyses of the average mitochondrial length for experiment C and E, respectively. The values represent the means ± SE of three independent experiments. *P < 0.05; **P < 0.01; ns, not significant.

Since different cancer cell types exhibit similar basic responses to TRAIL, we investigated the precise mechanisms that cause the mitochondrial network abnormalities by using melanoma cells and melanocytes as representatives of malignant cells and their normal counterpart, respectively. Then, we examined the effect of TRAIL on phosphorylation of Drp1 at Ser616, since this event is essential for mitochondrial fission [16]. Immunoblot analyses using an antibody directed against Drp1 phosphorylated at Ser616 (pDrp1 Ser616) indicated that TRAIL treatment increased the level of pDrp1 Ser616. Figure 4A shows the representative blots using A375 cells. The level of pDrp1 Ser616 clearly first increased at 2 h, and thereafter keeping the level for at least 4 h (Figure 4A, top). On the other hand, the level of pDrp1 at Ser637, which inhibits mitochondrial fission [16], was first observed at 30 min, reaching the maximum at 1 h, and thereafter declining (Figure 4A, middle). We also checked the possible changes in the expression levels of Drp1, Fis1, and Mfn1 and found that their expression levels were marginally changed (Figure 4B). To determine whether the pro-apoptotic mitochondrial network abnormalities were attributed to the Drp1 pathway, we analyzed the effect of TRAIL on the mitochondrial network in A375 cells in which Drp1 protein levels were down-regulated by siRNA interference as previously reported [26]. Compared to control cells treated with irrelevant scrambled siRNA, cells treated with siRNA targeting Drp1 exhibited significantly elongated mitochondrial network, indicating that mitochondrial fission is impaired in these cells (Figure 4C, lower left and 4D). Nevertheless, the Drp1 knockdown significantly enhanced TRAIL-induced mitochondrial fragmentation and clustering (Figure 4C, lower right). As a result, the average mitochondrial length became significantly shorter than that observed in control cells treated with TRAIL (Figure 4D). To verify these observations, we also examined the effect of mdivi-1, a potent Drp1 inhibitor, which can inhibit mitochondrial fission in multiple human cancer cell types and sensitizes them to TRAIL-induced apoptosis [26]. Mdivi-1 treatment elongated mitochondria in some cells while it promoted modest mitochondrial fragmentation in others. Totally, no significant increase in the average mitochondrial length was observed compared to non-treated control cells (Figure 4E, lower left, 4F). However, similar to Drp1 knockdown, Mdivi-1 treatment significantly accelerated TRAIL-induced mitochondrial fragmentation and clustering (Figure 4E, lower right), resulting in mitochondria with the average length of ∼ 2 μm (Figure 4F). Collectively, these results show that although TRAIL induces Drp1-dependent mitochondrial fission, this process inhibits rather than mediates mitochondrial network abnormalities.

### Correlation among depolarization, mitochondrial dysfunction, and apoptosis

**Figure 5:**
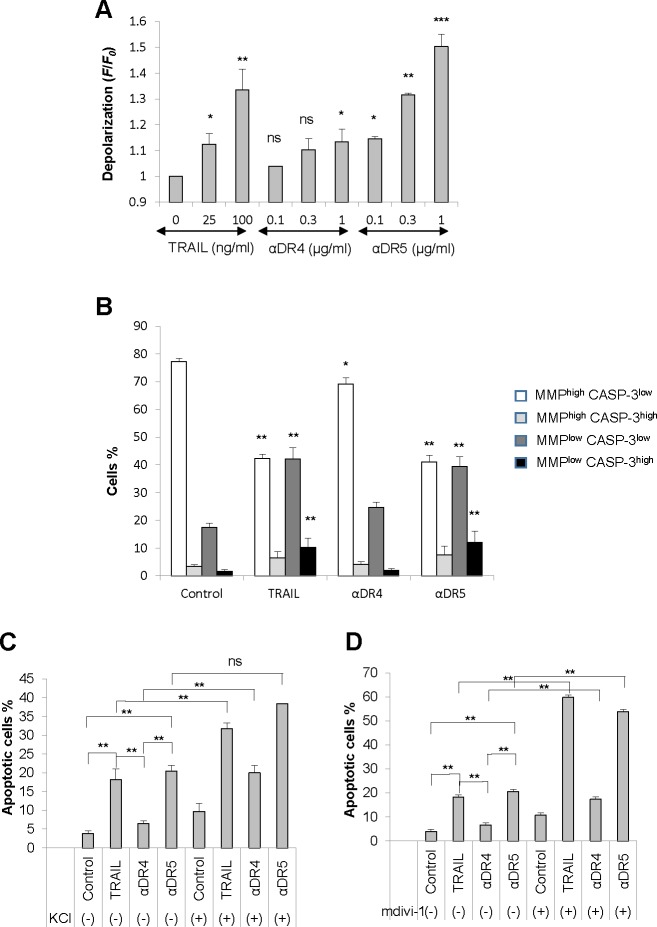
Correlation among depolarization, mitochondrial dysfunction, and apoptosis **A.** A375 cells were treated with TRAIL, αDR4, or αDR5 at the indicated concentrations for 4 h, and analyzed for cell membrane depolarization using *bis*-oxonol by flow cytometry. The data were expressed as the ratio of mean fluorescence intensity, *F*/*F*_0_, where *F*_0_ is the fluorescence in unstimulated cells and *F* is the fluorescence in stimulated cells and represent the means ± SE of three independent experiments. *P < 0.05; **P < 0.01; ***P < 0.001; ns, not significant. **B.** The cells were treated with TRAIL (100 ng/ml), αDR4, or αDR5 (1 μg/ml) for 24 h and analyzed for caspase-3/7 (CASP-3) activation and MMP collapse. The data were expressed as a percentage of each cell population in the histogram and the values represent the means ± SE of four independent experiments. **P < 0.01. **C.**, **D.** The cells were treated with TRAIL (100 ng/ml), αDR4, or αDR5 (1 μg/ml) alone or in combination with either KCl (50 mM, C) or mdivi-1 (50 μM, D) for 24 h, and then analyzed for annexin V/PI staining by flow cytometry. Annexin V^+^ cells were considered to be apoptotic cells. The values represent the means ± SE of four or five independent experiments. **P < 0.01; ns, not significant.

Considering that depolarization is a key pro-apoptotic event in TRAIL-induced apoptosis [12, 13], we hypothesized that it might play a role in the mitochondrial network abnormalities. To test this possibility, first we analyzed the capacity of the αDR4 and the αDR5 to evoke depolarization. As shown in Figure 5A, flow cytometric analyses using the anionic dye *bis*-oxonol showed that TRAIL and αDR5 dose-dependently and significantly induced depolarization, while αDR4 only modestly induced depolarization. Next, we analyzed the collapse of MMP and caspase-3 activation, two key events in the intrinsic pathway by flow cytometry. Based on their staining, cells were divided into four groups, MMP^low^ CASP-3^low^, MMP^low^ CASP-3^high^, MMP^high^ CASP-3^low^, or MMP^high^ CASP-3^high^ cells. In control cells, the majority of the cell population (77.3 ± 1.1%) was MMP^high^ CASP-3^low^, and other minor populations were MMP^low^ CASP-3^low^ (17.6 ± 1.4%), MMP^high^ CASP-3^high^ (3.5 ± 0.6%), or MMP^low^ CASP-3^high^ cells (1.7 ± 0.5%) (*N* = 4), indicating that MMP is intact and there is minimal caspase-3 activation (Figure 5B). Treatment with TRAIL resulted in a significant increase in MMP^low^ CASP-3^low^ cells up to 42.2 ± 3.9%, concomitant with a significant decrease in MMP^high^ CASP-3^low^ cells down to 42.3 ± 1.6%. In addition, MMP^low^ CASP-3^high^ and MMP^high^ CASP-3^high^ cells were increased up to 9.0 ± 3.0% and 6.5 ± 2.2%, respectively. This indicates that TRAIL induces significant MMP collapse and caspase-3 activation. Treatment with αDR5 induced similar effects, while the αDR4 caused only modest MMP depolarization and minimal caspase-3/7 activation. Moreover, TRAIL and αDR5 were similarly able to induce apoptosis, while αDR4 minimally induced apoptosis (Figure 5C, 5D). Although KCl and mdivi-1 alone had minimal effects, these two drugs significantly potentiated the pro-apoptotic effects of TRAIL and αDR5. They also significantly augmented αDR4-induced apoptosis to levels comparable to that induced by TRAIL and αDR5 alone. These results show the correlation among depolarization, mitochondrial dysfunction, and apoptosis.

### Depolarization potentiates the αDR4-induced mitochondrial network abnormalities

**Figure 6:**
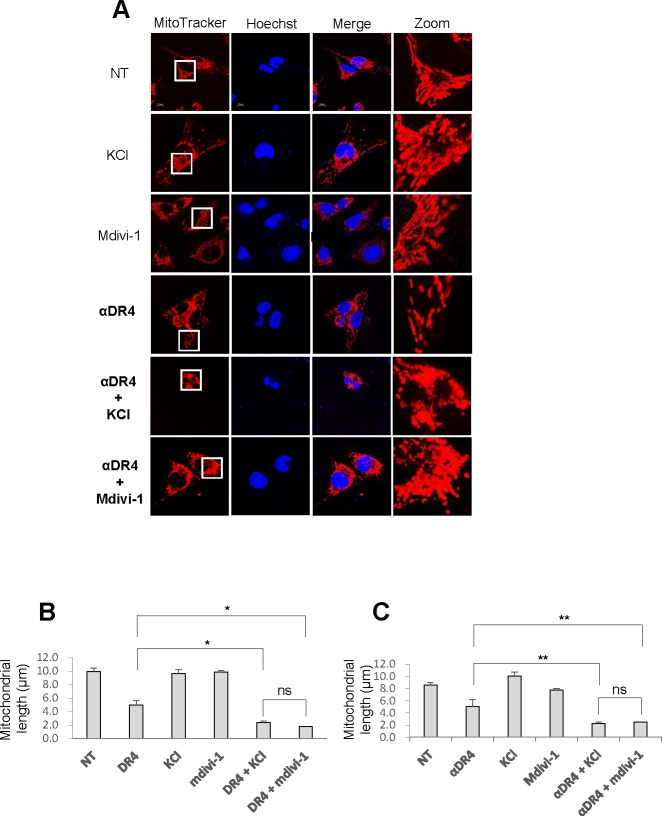
Depolarization potentiates αDR4–induced mitochondrial network abnormalities **A.** A2058 cells **A.** and A375 cells were treated with αDR4 (1 μg/ml) alone or in combination with either KCl (50 mM) or mdivi-1 (50 μM) for 24 h at 37 °C, and analyzed for mitochondrial morphology using the confocal microscope. **B.**, **C.** Statistical analyses of the average mitochondrial length for A2058 cells **B.** and A375 cells **C.**. The values represent the means ± SE of three independent experiments. *P < 0.05; **P < 0.01; ns, not significant.

Next, we examined whether depolarization affects the effects of αDR4 on the mitochondrial network. Figure 6A shows the representative confocal pictures in A2058 cells. Upon high K^+^ loading, some mitochondria became elongated while other became moderately fragmented, as observed after αDR4 treatment. However, when KCl and αDR4 were applied together, mitochondria primarily became punctate and clustered (Figure 6A). Moreover, these mitochondria harbored damaged or fragmented nuclei. Similar to KCl, mdivi-1 also potentiated αDR4-induced mitochondrial network abnormalities (Figure 6A). Judging from the mitochondrial morphology and the average mitochondrial length, Mdivi-1 and KCl potentiated the αDR4–induced mitochondrial network abnormalities to a similar extent (Figure 6B and 6C). Similar results were obtained with A375 cells (data not shown).

### Normal cells are resistant to DR-mediated depolarization, mROS accumulation, and mitochondrial network abnormalities

**Figure 7:**
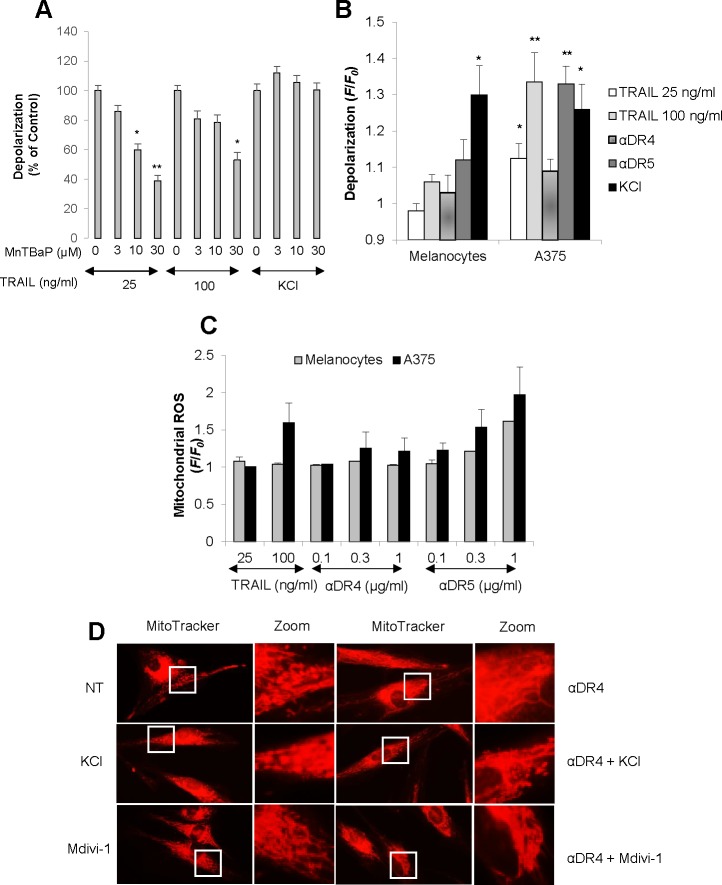
Minimal depolarization and mROS accumulation in normal melanocytes upon DR ligation **A.** A375 cells were treated with TRAIL at the indicated concentrations or KCl (50 mM) in the presence or absence of MnTBaP at the indicated concentrations for 4 h, and analyzed for cell membrane depolarization using *bis*-oxonol by flow cytometry. The data are shown as the percentages of the fluorescence intensity relative to control cells stimulated with TRAIL alone (set at 100%) and represent the means ± SE of four independent experiments. *P < 0.05; **P < 0.01. **B.** A375 cells and melanocytes were treated with TRAIL (25, 100 ng/ml), αDR4, or αDR5 (1 μg/ml), or with KCl (50 mM), and analyzed for cell membrane depolarization. The data were expressed as the ratio of mean fluorescence intensity, *F*/*F*_0_, where *F*_0_ is the fluorescence in unstimulated cells and *F* is the fluorescence in stimulated cells and represent the means ± SE of three or four independent experiments. *P < 0.05; **P < 0.01. **C.** A375 cells and melanocytes were treated with TRAIL (100 ng/ml) or the indicated concentrations of αDR4, or αDR5 for 4 h, and analyzed for mitochondrial ROS accumulation. The values represent the means ± SE of three independent experiments. **D.** HDF cells were treated with αDR4 (1 μg/ml) in the absence or presence of KCl (50 mM) or mdivi-1 (50 μM) for 24 h, and then analyzed for mitochondrial morphology. Representative pictures of three independent experiments with similar results were shown.

Our earlier studies show that depolarization and mROS mutually regulate one another during TRAIL-induced apoptosis [13, 28]. Therefore, we determined whether the TRAIL-induced depolarization in melanoma cells was also ROS-dependent. Treatment with MnTBaP, a superoxide dismutase-mimetic at concentrations ranging from 3 to 30 μM dose-dependently reduced TRAIL-induced depolarization in A375 cells, although the effects of MnTBaP were usually more pronounced for TRAIL (25 ng/ml) than for TRAIL (100 ng/ml) (Figure 7A). As a result, MnTBaP (30 μM) inhibited the depolarization induced by TRAIL (25 ng/ml) by 61.1 ± 3.7% (*N* = 3). On the other hand, depolarization induced by high K^+^ loading was minimally affected by MnTBaP treatment (Figure 7A). These results show that TRAIL specifically induces depolarization in a ROS-dependent manner in these cells. Next, we analyzed the ability of TRAIL to induce depolarization in melanocytes in which TRAIL induces minimal pro-apoptotic mitochondrial network abnormalities. TRAIL, αDR5 and αDR4 all induced minimal depolarization in melanocytes, while high K^+^ loading caused substantial depolarization comparable to that observed in A375 cells (Figure 7B), indicating that melanocytes are specifically resistant to TRAIL-induced depolarization. Since mROS appears to regulate depolarization, we next compared mROS generation between these two cell types using MitoSOX Red. This dye localizes to mitochondria and serves as a fluoroprobe for selective detection of superoxide in these organelles [29, 30]. Consistent with our earlier study [28], TRAIL treatment substantially increased mROS levels in A375 cells (Figure 7C). αDR5 also dose-dependently increased mROS levels while αDR4 had minimal effect. In contrast, all of these agents only modestly increased mROS levels in melanocytes. We also found that unlike tumor cells, minimal punctate mitochondria and their clustering were observed in normal cells even when αDR4 was applied together with KCl or Mdivi-1 (Figure 7D). Collectively, these findings suggest that normal cells are more resistant than tumor cells to DR-mediated depolarization, mROS accumulation, and mitochondrial network abnormalities.

## DISCUSSION

In the present study, we show that TRAIL exerts distinct effects on the mitochondrial networks in malignant cells and normal cells. TRAIL induced heavy fragmentation of mitochondria into punctate and their clustering in multiple human cancer cell lines (Figures 1, 2A) and predominantly caused moderate fission in normal cells such as melanocytes and fibroblasts (Figure 2B). Analyses using agonistic αDR4 and αDR5 antibodies indicated that these effects are attributed to DR ligation (Figure 3A, 3B). Considering that TRAIL induced apoptosis in cancer cells, but not in normal cells, it is possible that moderate fission is adaptive while mitochondrial fragmentation and clustering are pro-apoptotic. Consistent with this view, the clustering of punctate mitochondria was specifically associated with cell damage as well as nuclear fragmentation and chromatin condensation, the hallmarks of apoptosis (Figures 3E, 6A). Moreover, it is likely that the punctate mitochondria results from mitochondrial swelling, a consequence of mitochondrial dysfunction and integrity collapse. In addition, the observation that αDR4 lacking the pro-apoptotic activity (Figure 5C, 5D) induced moderate fragmentation, but not clustering of punctate mitochondria, also supports the pro-apoptotic role of the latter response (Figure 3A, 3B). Previous studies have shown that mitochondrial clustering precedes cytochrome c release during etoposide-, anti-Fas- or arsenic trioxide-induced apoptosis in leukemia cells [31, 32]. The authors also have shown that mitochondrial clustering occurs upstream of caspase-3 activation, and is specifically associated with intrinsic death pathway, suggesting the pro-apoptotic role. Hyperfusion of mitochondria caused by fission inhibition is observed in different cancerous cells and in normal cells upon stimulation with diverse stimuli. Tondera et al. [33] have shown that in several cell types including mouse embryonic fibroblasts (MEFs), mitochondria hyperfuse to form highly-interconnected networks in response to a number of apoptotic stimuli such as UV irradiation, actinomycin D and cycloheximide. This stress-induced mitochondrial hyperfusion (SIMH) requires OPA1, MFN1 and the mitochondrial inner membrane protein, stomatin-like protein 2 (SLP-2), and is adaptive to metabolic insults. SIMH is a pro-survival response against stress-induced apoptosis, as evidenced by the higher sensitivity of cells deficient in MFN1 or SLP-2 to actinomycin D and UV irradiation. Moreover, enforced mitochondrial hyperfusion, due to the expression of dominant-negative mutant of Drp1 or membrane-associated ring finger 5, promotes NF-B activation, and the expression of anti-apoptotic genes such as Bcl-XL, X-linked inhibitor of apoptosis protein, and FLICE-inhibitory protein, in human embryonic kidney cells (HEK293), HeLa cells, and MEFs [34]. On the other hand, mitochondrial hyperfusion induced by knockdown of Drp1 or mitochondrial fission factor, delays cell cycle progression and promotes G2/M accumulation and caspase-dependent cell death in U2OS osteosarcoma cells [35]. These observations suggest that mitochondrial hyperfusion is pro-apoptotic. Thus, at present the role of mitochondrial hyperfusion in cancer cell apoptosis is controversial. Consistent with our previous study [26], Drp1 knockdown and mdivi-1 treatment increased elongated mitochondria in tumor cells (Figure 4C-4E). However, unlike Drp1 knockdown, we also observed modest mitochondrial fragmentation in some mdivi-1 treated cells (Figures 4E, 6A). Totally, we observed no significant increase in the average mitochondrial length (Figure 4F). Similar dual effects were observed in KCl treated cells (Figure 6A). In our systems, mitochondrial network abnormalities occurred sequentially; mitochondrial truncation associated with local mitochondrial elongation was first observed at early time points (30 min) after TRAIL treatment while mitochondrial fragmentation and clustering regularly became prominent only at late time points (4-24 h) (Figure 1B). Moreover, while αDR4 or KCl alone induced mitochondrial truncation and local mitochondrial elongation, their combined application caused substantial mitochondrial fragmentation and clustering. This suggests that the two modes of mitochondrial network abnormalities are not independent events but possibly are related. Collectively, it is possible that the dual response is either pro-survival or pro-apoptotic depending on its duration time or amplitude. Interestingly, mitochondrial hyperfusion like SIMH, may inherently be an adaptive, pro-survival response, which may be restored thereafter while mitochondrial hyperfusion may act as a pro-apoptotic response when it lasts persistently, as reported by Westrate et al [35]. Consistent with the impact on mitochondrial network, TRAIL induced the phosphorylation of Drp1 at Ser616 or Ser637. Moreover, the duration time for which the phosphorylated proteins persisted was often different; pDrp1 Ser616 lasted for at least 4 h while pDrp1 Ser637 declined within 2 h (Figure 4A). Since pDrp1 Ser616 is essential for mitochondrial fission while pDrp1 Ser637 inhibits it [16], this may provide a bias toward mitochondrial fission over mitochondrial fusion. In fact, mitochondrial fragmentation and clustering progressed at late timing after TRAIL treatment. Given that mitochondrial hyperfusion results from mitochondrial fission inhibition, it might play a role in TRAIL-induced cell death, although we can observe only local mitochondrial elongation in some cells. Further investigation is necessary to determine this view.

Since mitochondrial fragmentation and clustering were accelerated rather than inhibited by Drp1 knockdown and by mdivi-1 treatment, Drp1-dependent mitochondrial fission may counteract the pro-apoptotic mitochondrial network abnormalities. This is also in accordance with our earlier observations that these two interventions potentiate TRAIL-induced mitochondrial dysfunctions and apoptosis [26]. Collectively, these observations suggest that the mitochondrial fragmentation observed here may be different from the reversible Drp1-dependent mitochondrial fission and may be irreversibly committed to mitochondrial dysfunction.

It is noted that mitochondrial fragmentation can occur in a Drp1-independent manner. Dimmer and colleagues have recently shown that down-regulation of LETM1, an inner mitochondrial membrane protein, leads to Drp1-independent fragmentation and clustering of mitochondria and cell death [36]. This event is not caused by an imbalance in the fission-fusion equilibrium but is related to ion homeostasis disruption, since LETM1 functions as K^+^/H^+^ antiporter. This is interesting when considering that TRAIL-induced mitochondrial fragmentation and clustering is augmented by depolarization, a major cause of ion homeostasis disruption and associated with cell death. Considering the similarity in their observations and ours, it is possible that similar Drp1-independent ion homeostasis-mediated mechanisms underlie in the TRAIL-mediated mitochondrial fragmentation. Further studies on the possible role of LETM1 and ion homeostasis disruption in the mitochondrial fragmentation in the pro-apoptotic mitochondrial abnormalities are currently underway.

Another important finding in this study is that persistent depolarization is required for the pro-apoptotic mitochondrial network abnormalities, though depolarization *per se*, did not cause mitochondrial fragmentation and clustering (Figure 6A). αDR4, which could induce minimal mitochondrial fragmentation and clustering (Figures 3A, 3C, 3E, 6A), also induced minimal depolarization together with minimal mitochondrial dysfunction, caspase-3 activation, and apoptosis (Figure 5A–5D). However, in the presence of KCl or mdivi-1, αDR4 induced substantial mitochondrial fragmentation and clustering (Figure 6A). In addition, a considerable level of apoptosis, comparable to that induced by TRAIL or αDR5 alone, was observed under these conditions, while KCl or mdivi-1 alone caused minimal apoptosis (Figure 5C, 5D). These results suggest that depolarization plays a pivotal role in the progression from non-apoptotic to the pro-apoptotic mitochondrial network abnormalities, though further studies are necessary to elucidate the mechanisms by which depolarization elicits these effects.

It may be worthwhile to determine the molecular basis of the tumor-specific mitochondrial network abnormalities, because central targeting can be exploited to enhance the induction of tumor-targeting apoptosis. In expansion of our earlier work using Jurkat leukemia cells [13], we found that mROS also contributed to TRAIL-induced depolarization in melanoma cells (Figure 7A). More importantly, DR ligation preferentially induced depolarization and mROS accumulation in malignant cells over normal cells (Figure 7B, 7C)

**Figure 8:**
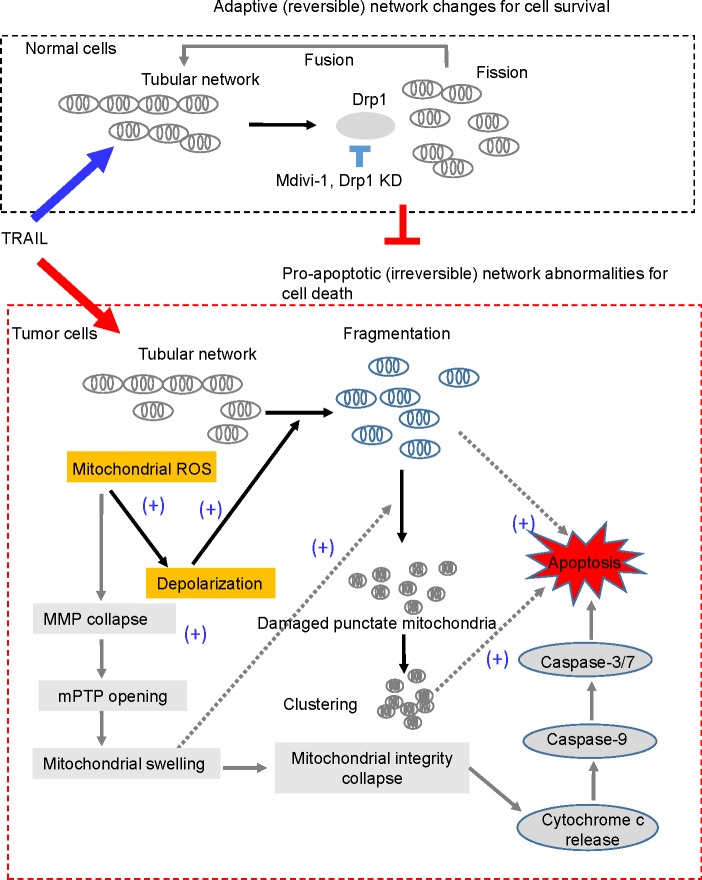
A hypothetical model for the tumor-selective mitochondrial network abnormalities induced by TRAIL In response to DR agonists such as TRAIL, tubular mitochondria in normal cells undergo Drp1-dependent fission and then fuse to restore the tubular network (upper panel). This may make normal cells resistant to the stress. On the other hand, in tumor cells, relatively higher levels of mitochondrial ROS (mROS) accumulation occur and then mROS evoke depolarization, which promotes mitochondrial fragmentation and clustering. Consequently, a smaller number of mitochondria can restore the tubular network through the fission-fusion homeostasis described above. Hence, this mitochondrial morphology homeostasis can be anti-apoptotic by counteracting pro-apoptotic (irreversible) mitochondrial network abnormalities, impairment of the Drp1-dependent fission by Drp1 knockdown or mdivi-1 accelerates mitochondrial fragmentation and clustering, and cell death. mROS accumulation also causes mitochondrial membrane potential (MMP) collapse, resulting in the opening of the mitochondrial permeability transition pore (mPTP), mitochondrial swelling and collapse of mitochondrial integrity, a central gatekeeper of the intrinsic apoptosis pathway. Mitochondrial swelling and depolarization may cooperatively facilitate the formation and clustering of damaged punctate mitochondria. Either or both of mitochondrial fragmentation and clustering may promote apoptotic cell death.

Collectively, our results imply that higher depolarization and mROS accumulation in malignant cells cause the tumor-selective mitochondrial network abnormalities, leading to apoptosis (Figure 8).

In summary, we demonstrate in this paper that TRAIL induces the mitochondrial network abnormalities associated with apoptotic cell death in human malignant cells, but not in normal cells and that depolarization plays a critical role in this process. To our knowledge, these findings are the first to show that TRAIL affects the mitochondrial network in human malignant cells and may provide insight into the molecular basis of the tumor-targeting killing effect of TRAIL.

## MATERIALS AND METHODS

### Reagents

Soluble recombinant human TRAIL and mdivi-1 were obtained from Enzo Life Sciences (San Diego, CA). Anti-human TRAIL-R4 (clone 104918 #MAB633) and anti-human TRAIL-R5 (clone 71903 #MAB631) were purchased from R&D systems (Minneapolis, MN). Mouse anti-Drp1 monoclonal antibody (C-5, #sc-271583), goat polyclonal anti-Fis1 antibody (K-14, sc-48865), and mouse monoclonal anti-Mfn1 antibody (D-10 sc-166644) were obtained from Santa Cruz Biotechnology (Dallas, TX).

### Cell culture

The human melanoma cell lines, A549 cells (human lung adenocarcinoma epithelial cell line), and osteosarcoma cell lines were obtained from Health Science Research Resource Bank (Osaka, Japan). Human dermal fibroblasts from facial dermis were obtained from Cell Applications (San Diego, CA). These cell lines were cultured in Dulbecco's modified Eagle's medium (DMEM; Sigma-Aldrich) supplemented with 10% fetal bovine serum (FBS; Sigma-Aldrich) (FBS/DMEM) in a 5% CO_2_ incubator. Normal human epidermal melanocytes were obtained from Cascade Biologics (Portland, OR), and cultured in DermaLife Basal Medium supplemented with DermaLife M LifeFactors (Kurabo, Osaka, Japan). Cells were harvested by incubation in 0.25% trypsin-EDTA (Life Technologies Japan) for 5 min at 37°C.

### Apoptosis

Apoptotic cell death was quantitatively assessed by double-staining with fluorescein isothiocyanate (FITC)-conjugated annexin V and propidium iodide (PI) as previously described [12]. Briefly, cells (2 × 10^5^/well) in 24-well plates were incubated with the agents to be tested for 24 h in FBS/DMEM at 37°C. Subsequently, the cells were stained with FITC-conjugated annexin V and PI using a commercially available kit (Annexin V FITC Apoptosis Detection kit I; BD Biosciences Japan). The stained cells were evaluated in the FACSCalibur (BD Biosciences Japan) and analyzed using CellQuest software (BD Biosciences). Four cellular subpopulations were evaluated: viable cells (annexin V^−^/PI^−^); early apoptotic cells (annexin V^+^/PI^−^); late apoptotic cells (annexin V^+^/PI^+^); and necrotic/damaged cells (annexin V^−^/PI^+^). Annexin V^+^ cells were considered to be apoptotic cells.

### Mitochondrial network imaging acquisition and length measurements

The mitochondrial network was analyzed by staining with the mitochondria-targeting dye MitoTracker^®^ Red CMXRos (Life Technologies Japan) as previously described [26] with minor modifications. Briefly, cells in FBS/DMEM were placed at the density of 5 × 10^4^/300 μl/well on an 8-well chambered coverglass (Thermo Fisher Scientific, Rochester, NY) and treated with the agents to be tested for 24 h at 37°C in a 5% CO_2_ incubator. After removing the medium by aspiration, the cells were washed with Hank's balanced salt solution (HBSS), and stained with 20 nM MitoTracker Red CMXRos and Hoechst 33342 (Dojindo) in HBSS for 1 h at 37°C in the dark in a 5% CO_2_ incubator. The cells were then washed with and immersed in FluoroBrite^TM^ DMEM. Images were obtained and analyzed using an EVOS FL Cell Imaging System (Life Technologies Japan) equipped with digital inverted microscope at ×1200 magnification. MitoTracker Red and Hoechst 33342 signals were captured using Texas Red and DAPI Light Cubes, respectively. For confocal imaging, samples were observed using a laser scanning microscopy with Airyscan (LSM 700 and 880, Carl-Zeiss microscopy Japan, Tokyo, Japan) equipped with a oil-immersion objective (Plan Apochromat 63x/1.4; Carl Zeiss) with 555/561 and 405 nm laser. Images were enlarged and analyzed for mitochondrial length using the free NIH ImageJ software (NIH, USA) and ZEN lite 2012 (Carl-Zeiss).

### Depolarization analysis

Depolarization was measured by flow cytometry using *bis*-oxonol, an anionic dye that increases in fluorescence intensity upon membrane depolarization, as previously described [12]. Briefly, cells (4×10^5^ cells/500 μl) suspended in HBSS were incubated with 100 nM dye for 15 min at 37°C, and then incubated with the agents to be tested for 4 h at 37°C in a 5% CO_2_-containing atmosphere. Subsequently, 1×10^4^ cells were counted for their fluorescence using the FL-2 channel of a FACSCalibur and analyzed using the CellQuest software. The data were expressed as *F*/*F*_0_, where *F*_0_ is the fluorescence in unstimulated cells and *F* is the fluorescence in stimulated cells.

### Caspase-3/7 activation and MMP collapse

Activation of caspase-3/7 and MMP collapse were simultaneously measured by flow cytometry as previously described [12]. Briefly, cells (2 × 10^5^/ml) in 24-well plates were treated with the agents to be tested for 24 h in 10% FBS/DMEM at 37°C, and then stained with the dual sensor MitoCasp^TM^ kit (Cell Technology, Mountain View, CA). Caspase-3/7 activation and MMP were evaluated using FACSCalibur according to the manufacturer's instructions, and the data were analyzed using CellQuest software.

### mROS

mROS levels were measured using MitoSOX^TM^ Red (Life Technologies Japan) by flow cytometry and the signals were calibrated as previously described [28]. Briefly, cells (5 × 10^5^ /500 μl) suspended in HBSS were incubated with the agents to be tested for 4 h at 37°C and then incubated with 5 μM MitoSOX for 15 min at 37 °C for loading. The cells were washed, resuspended in HBSS on ice, centrifuged at 4°C and then analyzed for their fluorescence using FACSCalibur. The data were analyzed using CellQuest software and expressed as *F*/*F_0_*, where *F_0_* is the fluorescence of unstimulated cells and *F* is the fluorescence of stimulated cells.

### Drp1 knockdown

Mitochondrial fission was inhibited by down-regulating Drp1 expression using small interfering RNA (siRNA) as previously described [26]. Cells (2.5 × 10^5^ /well) were plated in six-well plates and transfected with 20 nM of either Drp1-targeting siRNA (#sc-43732, Santa Cruz Biotechnology, Santa Cruz, CA) or scrambled control siRNA (#sc-37007, Santa Cruz) using Lipofectamine RNA/Max Kit (Life Technologies Japan) according to the manufacturer's instructions and cultured for 72 h at 37 °C in a 5% CO_2_ incubator.

### Immunoblotting

The level of Drp1 was determined by immunoblot analysis, as previously described [26]. The phosphorylation of Drp1 was assessed by immunoblotting. Briefly, cells (1.5 × 10^5^/ml) were washed twice with ice-cold PBS, lysed with RIPA buffer (Nacalai Tesque, Kyoto, Japan) containing protease inhibitors, and homogenized by sonication using a Bioruptor UCD-250 (Cosmo Bio, Tokyo, Japan). After centrifugation, the resulting supernatant was measured for protein content using a Pierce BSA Protein Assay Kit (Themo Fisher Scientific) according to the manufacturer's instructions. After heating at 70°C for 10 min, samples (15–20 μg protein) were subjected to reducing sodium dodecyl sulfate polyacrylamide gel electrophoresis (SDS-PAGE) using a 10% separation gel (Life Technologies) and transferred onto polyvinylidene difluoride membranes (Life Technologies). The membranes were blocked with Blocking One (Nacalai Tesque) for 1 h at room temperature, washed with Tris-buffered saline containing 0.1% Tween 20 (TBS-T), and then incubated with primary antibody; phosphor-Drp1 (Ser616; #3455) or phosphor-Drp1 (Ser637; #4867) (Cell Signaling Technology Japan, Tokyo, Japan) overnight at 4 °C. After washing with TBS-T, the membranes were incubated with secondary antibody for 1 h at room temperature. The signal was detected with the ECL Prime Western Blotting Detection Reagent (GE Healthcare, Little Chalfont, UK) using GAPDH (Abcam, Cambridge, UK) as a loading control.

### Statistical analysis

Data were analyzed by one-way analysis of variance followed by the post-hoc Tukey test using an add-in software for Excel 2012 for Windows (SSRI, Tokyo, Japan). All values were expressed as mean±SE, and *P* < 0.05 was considered to be significant.
